# Clinical characteristics and severity of influenza infections by virus type, subtype, and lineage: A systematic literature review

**DOI:** 10.1111/irv.12575

**Published:** 2018-07-20

**Authors:** Saverio Caini, Madelon Kroneman, Therese Wiegers, Clotilde El Guerche‐Séblain, John Paget

**Affiliations:** ^1^ Netherlands Institute for Health Services Research (NIVEL) Utrecht The Netherlands; ^2^ Global Health Economics and Outcomes Research Sanofi Pasteur Lyon France

**Keywords:** clinical presentation, influenza, literature review, severity, virus type

## Abstract

**Aim:**

Studies carried out in the early 2000s found that the number of influenza‐associated hospitalizations and deaths was highest in seasons dominated by A(H3N2), suggesting that the clinical presentation and severity of influenza may differ across virus types, subtypes, and lineages. We aimed to review the studies that examined this hypothesis.

**Method:**

We conducted a literature review of studies published until January 2017 that compared the clinical presentation, disease severity, and case‐fatality ratio of influenza patients infected with different virus types (A, B), subtypes (pre‐pandemic A(H1N1), A(H1N1)p, A(H3N2)), and lineages (Victoria, Yamagata).

**Results:**

The literature search resulted in over 1700 entries: After applying in‐ and exclusion criteria, 47 studies were included in the literature review. Studies showed a wide diversity in setting and populations. Only a minority of studies provided results adjusted by patient's age and other potential confounders. There were very few differences in the clinical presentation of patients infected with different influenza viruses. We found weak evidence that the A(H1N1)p subtype in the post‐pandemic period was more often associated with secondary bacterial pneumonia, ICU admission, and death, than the other influenza virus (sub)types.

**Conclusion:**

Contrary to what is commonly assumed, the causal virus subtype does not seem to be a major determinant of clinical presentation and severity of influenza illness. However, drawing conclusions was made difficult by the low comparability and methodological shortcomings of included studies, and more well‐designed studies are warranted.

## 1 INTRODUCTION

Influenza illness is clinically characterized by non‐specific signs and symptoms that are common to other respiratory infections, such as sudden onset, fever, malaise, headache, and cough.^1^ Influenza illness is usually short‐lived (3‐5 days), and severe outcomes are rare unless the person is elderly or has an underlying disease (such as chronic heart disease, diabetes, and cancer), a weakened immune system, or other medical condition. Influenza was described as “an unvarying disease caused by a varying virus” in 1975,^2^ suggesting that the illness caused by the different virus types and subtypes is clinically indistinguishable, but this has been challenged in recent years. Two ground‐breaking studies published by Thompson et al in 2003 and 2004 found that the number of hospitalizations and influenza‐associated deaths in the United States was highest during seasons in which A(H3N2) was the dominant subtype among the circulating viruses, followed by seasons in which influenza B or influenza A(H1N1) was dominant, and this was confirmed in later studies.^3^, ^4^, ^5^ Although these studies were not based on individual‐level clinical data but modeled data with aggregated national mortality, hospital discharge, and viral surveillance data, they have led to the hypothesis that the clinical presentation, severity, and risk of unfavorable outcomes of influenza illness may indeed differ across virus types and subtypes.

In recent years, the hypothesis that influenza severity is dependent on the causal virus type and subtype has been examined in several studies,^6^, ^7^, ^8^, ^9^, ^10^, ^11^, ^12^ which differed considerably between one another in terms of study setting and design, populations being examined, sample size, influenza viruses being compared, and ability to control for potential confounders (eg, patient's age, underlying comorbidities, and other predictors of disease severity and outcome). To our knowledge, no systematic review has been carried out to date that has attempted to summarize the available evidence, yet this question is of considerable importance from both a clinical and public health perspective, as it may have implications for the management of influenza patients, for communication and preparedness during seasonal epidemics (eg, regarding the number of influenza‐related hospitalizations to be expected during the influenza season), and for producing accurate cost‐benefit estimates of influenza vaccination campaigns and other prevention and control strategies. To help clarify this issue, we conducted a systematic review of published studies that compared the clinical presentation, course severity, and case‐fatality ratio of influenza patients infected with different virus types, subtypes and lineages.

## 2 METHODS

### 2.1 Literature search and inclusion criteria

We searched articles in MEDLINE using the following search string: influenza AND (sign(s) OR symptom(s) OR clinical OR comorbidity OR severity OR complication(s) OR death) AND (comparison OR compare/s/d). We considered all papers published until January 31, 2017, that were written in English or in another language mastered by at least one study researcher (ie, French, Spanish, Italian, or Dutch). Two study researchers independently carried out an initial screening of all entries based on their title and abstract: Papers that were considered eligible for the review were obtained and read in full copy text format. In the next step, the eligibility of each paper was independently assessed by two study researchers; any disagreements were resolved via consensus. Papers were considered to be eligible for inclusion if they compared the clinical presentation (signs and symptoms), the presence of underlying conditions, or the disease severity (eg, complications, hospitalization, admission to an intensive care unit [ICU], need for ventilation support, or case‐fatality ratio) between laboratory‐confirmed influenza patients infected with different influenza virus types (A, B), subtypes (pre‐pandemic A(H1N1), A(H1N1)p, A(H3N2)), and lineages (Victoria, Yamagata). We excluded studies in which all included influenza cases were infected with only one influenza virus (sub)type, those focusing on avian influenza viruses, and those that were carried out during the pandemic period (ie, all patients were enrolled between April 2009 and July 2010). The references of all retrieved papers were tracked to find additional publications.

### 2.2 Data extraction

Data were extracted from each article by one study researcher, entered into a database expressly developed for the project, and independently cross‐checked by a second study researcher. In addition to main outcomes, we extracted information on factors that were considered to be relevant for the correct interpretation of the results, namely:

1. Country, region, and years in which the study was conducted;
2. Study setting and criteria for inclusion of laboratory‐confirmed influenza patients (eg, patients reported to community‐based surveillance system, individuals visiting the emergency room of hospitals and clinics, inpatients), and whether the study was conducted among specific population subgroups (eg, asthma patients, healthcare personnel, pregnant women);
3. Definition of influenza‐like illness, acute respiratory infection, and/or severe acute respiratory infection;
4. Number of virologically confirmed influenza cases, broken down by virus type, subtype, and lineage;
5. Age, gender, vaccination status of laboratory‐confirmed influenza patients and use of antivirals, and whether the reported results were adjusted by these variables;
6. Definition of each sign and symptom, underlying conditions, illness severity, complications, and of all the other outcomes being compared;
7. Statistical methods and variables used to adjust estimates (if any).

### 2.3 Assessment of the quality of studies

For observational studies, such as the studies included in our systematic review, several quality assessment tools or grids exist,^13^ many of which are, however, specifically developed for studies with a case‐control or cohort design. Considering most of the studies included in our review have a cross‐sectional design, we opted to score all included studies using a slightly modified version of the Quality Assessment Tool for Observational Cohort and Cross‐Sectional Studies developed by the National Heart, Lung and Blood Institute,^14^ which is an adequate tool to assess the quality of the studies and the risk of bias.

### 2.4 Statistical analysis

The main characteristics of all selected studies are reported in Tables 1 and 2. The studies were divided into two groups: studies in which all included influenza cases were treated as inpatients (ie, hospital‐based studies) and studies in which only a subset of patients were eventually hospitalized (these included community‐based studies, studies in which patients were enrolled among those visiting the emergency room of a hospital, and others). This was done based on the expectation that results may differ when all patients are hospitalized, because these patients may be more severely ill compared to patients from settings that include outpatients or are community‐based patients.

**Table 1:** Main characteristics and number of influenza patients by virus type, subtype, and lineage, of hospital‐based studies included in the review

First author, y	Country	Study period	Age group or range	A	B	A(H1N1)	A(H1N1)p	A(H3N2)	B Victoria	B Yamagata	Population studied (syndrome)
Children											
Weigl, 2002^18^	Germany	1996‐2001	≤16	122	14	‐	‐	‐	‐	‐	ARI
Dawood, 2011^19^	USA	2003‐09	≤17	‐	116	‐	733^a^	494	‐	‐	Children with asthma
Chiu, 2011^20^	China	2009	<18	‐	‐	99	99^a^	99	‐	‐	Respiratory symptoms
Daley, 2000^15^	Australia	1997	Children	64	27	‐	‐	‐	‐	‐	Any virus isolation from nasopharyngeal aspirates
Hu, 2004^21^	Taiwan	2000‐01	Children	73	124	‐	‐	‐	‐	‐	Not specified
Meury, 2004^22^	Switzerland	2001‐02	Children	45	15	‐	‐	‐	‐	‐	Respiratory symptoms
Guan, 2015^23^	China	2010‐12	Children	‐	59	‐	26	131	‐	‐	Lower RTI
Mancinelli, 2016^24^	Italy	2012‐13	Children	‐	‐	‐	54	8	6	65	RTI
Adults											
Yang, 2014^25^	China	2010‐11	≥14	‐	‐	‐	58	30	‐	‐	Pneumonia
Jennings, 2008^26^	New Zealand	1999‐2000	≥18	23	6	‐	‐	‐	‐	‐	Community‐acquired pneumonia
Loubet, 2016^27^	France	2012‐15	≥18	422	144	‐	163	239	‐	‐	ILI
Seo, 2014^28^	Korea	2009‐12	Adults	55	31	‐	‐	‐	‐	‐	ARI
Drinka, 1999^17^	USA	1988‐99	Elderly^b^	322	129	‐	‐	‐	‐	‐	ARI
All ages											
Rahamat‐Langendoen, 2012^29^	The Netherlands	2007‐11	All ages	‐	50	45	85^a^	‐	‐	‐	ARI
Chaves, 2013^9^	USA	2010‐11	All ages	‐	948	‐	924	1749	‐	‐	Not specified
Cohen, 2014^30^	South Africa	2009‐12	All ages	‐	418	‐	338^a^	463	‐	‐	SARI
Sočan, 2014^31^	Slovenia	2010‐13	All ages	‐	‐	‐	‐	‐	150	114	Lower RTI
Ishiguro, 2016^32^	Japan	2002‐14	All ages	‐	42	‐	20^a^	34	‐	‐	Influenza‐associated pneumonia
Kusznierz, 2016^33^	Argentina	2013	All ages	‐	‐	‐	46	54	‐	‐	Not specified
Puig‐Barberà, 2016^34^	Four countries	2013‐14	All ages	‐	‐	‐	362	534	3	130	ILI
Puig‐Barberà, 2016^35^	Six countries	2014‐15	All ages	‐	‐	‐	121	1243	11	623	ILI
Tan, 2013^36^	China	2009‐10	All ages	‐	‐	‐	‐	‐	139	43	ARI or community‐acquired pneumonia

ARI, acute respiratory infection; ILI, influenza‐like illness; RTI, respiratory tract infection; SARI, severe acute respiratory infection.

a Pandemic detections (2009/2010 season only) were not included in the analysis.

b All study participants were nursing home residents.

**Table 2:** Main characteristics and number of influenza patients by virus type, subtype, and lineage, of studies that included only non‐hospitalized or both hospitalized and non‐hospitalized patients

First author, y	Country	Study period	Age group or range	A	B	A(H1N1)	A(H1N1)p	A(H3N2)	B Victoria	B Yamagata	Population studied (syndrome)	Health seeking setting
Children												
Silvennoinen, 2009^16^	Finland	2000‐02	≤13	286	58	‐	‐	‐	‐	‐	Respiratory infections	Community cohort
Esposito, 2011^12^	Italy	2009‐10	<14	‐	‐	126	389^a^	486	‐	‐	ILI	Referrals to a hospital emergency room
Esposito, 2011^37^	Italy	2008‐09	<14	1751	392	‐	‐	‐	‐	‐	ILI	Primary care pediatricians
Shen, 2008^38^	Taiwan	2005‐07	<16	151	123	‐	‐	‐	‐	‐	RTI or febrile illness	In‐ and outpatients
Peltola, 2003^39^	Finland	1980‐99	<17	544	139	‐	‐	‐	‐	‐	Not specified	In‐ and outpatients
Hite, 2007^40^	USA	2002‐03	<19	112	93	‐	‐	‐	‐	‐	ILI	In‐ and outpatients
Chi, 2008 7	Taiwan	2001‐06	Children	163	118	‐	‐	‐	8	54	ARI	In‐ and outpatients
Adults												
Gutiérrez‐Pizarraya, 2012^41^	Spain	2010‐11	>14	‐	50	‐	80	‐	‐	‐	Severe illness, pregnant women, transplant recipients	In‐ and outpatients
Wright, 1980^42^	USA	1977‐78	College students	‐	‐	28	‐	47	‐	‐	Lower or upper RTI	University student health service
Yap, 2012^43^	Singapore	2009‐10	Young adults	‐	269	‐	434^a^	58	‐	‐	Febrile respiratory illness	Military primary healthcare clinic
Kaji, 2003^6^	Japan	1999‐2001	Adults	‐	44	54	‐	98	‐	‐	Respiratory symptoms	University hospital
Wie, 2013^44^	Korea	2011‐12	Adults	‐	194	‐	‐	656	‐	‐	ILI	Emergency rooms and university hospitals
All ages												
Frank, 1985^45^	USA	1977‐82	All ages	‐	‐	126	‐	182	‐	‐	Respiratory symptoms	Community cohort
Belongia, 2010^46^	USA	2008‐09	All ages	‐	‐	221	545^a^	632	‐	‐	Patients with any of: fever, chills or cough	In‐ and outpatients
Tang, 2010^47^	Singapore	2009	All ages	‐	12	14	547^a^	167	‐	‐	Respiratory symptoms	Primary care clinics and hospital emergency department
Irving, 2012^8^	USA	2004‐08	All ages	901	284	‐	‐	‐	‐	‐	ARI	Population‐based study
Lindblade, 2010^48^	Guatemala	2008‐09	All ages	‐	‐	51	162^a^	21	‐	‐	ILI	Public hospital and ambulatory clinics
Suess, 2012^49^	Germany	2007‐11	All ages	‐	38	6	70^a^	8	‐	‐	ILI	General practitioners and pediatricians
Yang, 2012^50^	China	2009	All ages	‐	‐	117	265^a^	162	‐	‐	ARI	In‐ and outpatients
Kawai, 2013^51^	Japan	2009‐11	All ages	‐	93	‐	199	96	‐	‐	ILI	General practitioners, pediatricians, and physicians
Dangi, 2014^10^	India	2010‐12	All ages	‐	‐	‐	129	63	57	174	ILI or SARI	Not specified
Hayward, 2014^52^	England	2006‐11	All ages	‐	35	10	102^a^	35	‐	‐	ARI	Community cohort
Sočan, 2014^31^	Slovenia	2010‐13	All ages	‐	‐	‐	‐	‐	228	145	ILI	Outpatient clinics or regional hospitals
Cohen, 2015^53^	France and Turkey	2010‐12	All ages	355	419	‐	‐	‐	‐	‐	ILI or ARI	Predominantly visits to general practitioner
Hong, 2015^54^	Korea	2011‐12	All ages	477	332	‐	‐	‐	‐	‐	Respiratory symptoms	In‐ and outpatients
Mosnier, 2015^11^	France	2004‐13	All ages	10977	3446	945	4022^a^	4483	778	1257	ARI	General practitioners and pediatricians

ARI, acute respiratory infection; ILI, influenza‐like illness; RTI, respiratory tract infection; SARI, severe acute respiratory infection.

a Pandemic detections (2009/2010 season only) were not included in the analysis.

The studies differed in the statistical methods that were used to compare the clinical presentation and severity of influenza illness between patients infected with different virus (sub)types. Some studies presented a measure of relative risk (RR) (ie, odds ratio or risk ratio) calculated through regression models: These were reported in Table 3 (for signs and symptoms) and Table 4 (for underlying conditions, complications, and outcomes), along with the variables that were used for adjusting the RR estimates. We had initially planned to pool study‐specific RRs into a summary estimate using random‐effects meta‐analysis models; however, this was not possible because of the large diversity of studies in terms of settings, populations, and definitions (see Results).

**Table 3:** Relative risk of selected signs and symptoms among patients infected with different influenza virus types, subtypes, and lineages

First author, y	Setting	Age group or range	Fever^a^	Cough^b^	Rhinitis^c^	Sore throat	Headache	Dyspnoea^d^	GI symptoms^e^	Myalgia	Age‐adjusted
A vs. B											
Hite, 2007 40	Hosp	<19	ns & nr	ns & nr	ns & nr	ns & nr	ns & nr	ns & nr	ns & nr	**0.34 (0.13‐0.91)**	Unclear
A(H1N1)p vs. B											
Yap, 2012 43	Non‐hosp	Young adults	**0.51 (0.29‐0.92)**	**2.1 (1.25‐3.54)**	**0.54 (0.34‐0.86)**	**0.44 (0.24‐0.80)**	ns & nr	‐	‐	ns & nr	Yes
Gutiérrez‐Pizarraya, 2012 41	Non‐hosp	Adults	**2.5 (1.2‐5.4)**	‐	‐	‐	‐	‐	ns & nr	‐	Unclear
A(H3N2) vs. B											
Yap, 2012 43	Non‐hosp	Young adults	ns & nr	ns & nr	ns & nr	ns & nr	ns & nr	‐	‐	ns & nr	Yes
Cohen, 2014 30	Hosp	All ages	ns & nr	ns & nr	‐	‐	‐	ns & nr	‐	‐	Yes
A(H1N1)p vs. A(H3N2)											
Yap, 2012 43	Non‐hosp	Young adults	**0.33 (0.11‐0.99)**	ns & nr	ns & nr	ns & nr	ns & nr	‐	‐	ns & nr	Yes
Cohen, 2014 30	Non‐hosp	All ages	ns & nr	ns & nr	‐	‐	‐	ns & nr	‐	‐	Yes
Dangi, 2014 10	Non‐hosp	All ages	‐	‐	‐	**2.27 (1.22‐4.22)**	ns & nr	ns & nr	ns & nr	ns & nr	Unclear
B Victoria vs. B Yamagata											
Dangi, 2014 10	Non‐hosp	All ages	‐	‐	‐	ns & nr	ns & nr	**12.0 (2.4‐59.8)**	**9.6 (1.0‐19.4)**	ns & nr	Unclear
Sočan, 2014 31	Non‐hosp	All ages	1.6 (0.5‐5.1)	1.1 (0.4‐3.0)	0.6 (0.3‐1.1)	1.1 (0.6‐1.9)	0.7 (0.3‐1.4)	‐	1.1 (0.5‐2.3)	1.5 (0.9‐2.7)	Yes
	Hosp		0.7 (0.3‐1.6)	0.7 (0.3‐1.8)	‐	‐	‐	0.9 (0.3‐1.8)	‐	‐	Yes

Ns, not statistically significant; nr, not reported; Hosp, hospital‐based studies, that is, in which all included patients were hospitalized. Non‐hosp, studies that included both hospitalized and non‐hospitalized patients.

a Fever, high fever.

b Cough (not specified), dry cough.

c Rhinitis, rhinorrhea, coryza, running nose.

d Dyspnoea, wheezing, shortness of breath.

e Gastrointestinal (GI) symptoms not specified, vomiting, diarrhea.

Bold value indicates the significant findings (*p* < .05).

**Table 4:** Relative risk of selected underlying conditions, associated respiratory infections, hospitalization, in‐hospital complications, length of hospital stay, and mortality, among patients infected with different influenza virus types, subtypes, and lineages

First author, y	Setting	Age group or range	Underlying conditions	Associated respiratory infections	Hospitalization, in‐hospital complications, length of hospital stay, and mortality	Age‐adjusted
A vs B						
Hite, 2007 40	Non‐hosp	<19	‐	URTI: nr & ns	Hospitalization: nr & ns	Unclear
				LRTI: nr & ns	Mechanical ventilation: nr & ns	
					Length of hospital stay: nr & ns	
					Death: nr & ns	
Irving, 2012 8	Non‐hosp	All ages	‐	Pneumonia: 1.2 (0.5‐2.8)	Hospitalization: 1.2 (0.7‐2.4)	Yes
A(H1N1)p vs B						
Gutiérrez‐Pizarraya, 2012 41	Non‐hosp	>14	Chronic cardiovascular disease: 1.1 (0.4‐3.1)	Pneumonia: 1.5 (0.7‐3.3)	Hospitalization: 1.6 (0.8‐3.3)	Unclear
			Chronic respiratory disease: 1.1 (0.5‐2.4)		ICU admission: 1.9 (0.6‐5.7)	
			Diabetes mellitus: 0.2 (0.04‐1.2)		Mechanical ventilation: 1.2 (0.4‐3.7)	
			Chronic renal disease: 0.8 (0.3‐2.8)		Death: 1.7 (0.6‐5.2)	
			Obesity: 0.9 (0.4‐2.3)			
Dawood, 2011 19	Hosp	≤17	Any underlying condition: **2.0 (1.4‐3.1)**	Pneumonia: 1.3 (0.8‐2.0)	ICU admission: 1.6 (0.9‐2.8)	Unclear
A(H3N2) vs B						
Chaves, 2013 9	Hosp	<18	‐	‐	ICU admission or death: 1.01 (0.56‐1.82)	Yes
Chaves, 2013 9	Hosp	Adults	‐	‐	ICU admission or death: 0.91 (0.65‐1.27)	Yes
Wie, 2013 44	Non‐hosp	Adults	‐	‐	Hospitalization: 1.19 (0.59‐2.40)	Yes
Cohen, 2014 30	Hosp	All ages	Any underlying condition: nr & ns	‐	ICU admission: nr & ns	
					Mechanical ventilation: nr & ns	
					Oxygen supplementation: nr & ns	
					Length of hospital stay: **0.62 (0.45‐0.83)**	
					Death: nr & ns	
A(H1N1) vs A(H1N1)p						
Esposito, 2011^12^	Non‐hosp	<14	‐	URTI: 0.88 (0.66‐1.16)	Hospitalization: **0.82 (0.59‐0.97)**	Yes
				LRTI: **0.62 (0.39‐0.99)**	Length of hospital stay: **0.18 (0.06‐0.56)**	
A(H1N1) vs A(H3N2)						
Esposito, 2011^12^	Non‐hosp	<14	‐	URTI: 0.75 (0.50‐1.14)	Hospitalization: **0.70 (0.51‐0.96)**	Yes
				LRTI: **0.59 (0.37‐0.93)**	Length of hospital stay: **0.41 (0.15‐0.65)**	
A(H1N1)p vs A(H3N2)						
Esposito, 2011^12^	Non‐hosp	<14	‐	URTI: 0.86 (0.43‐1.76)	Hospitalization: 0.96 (0.64‐1.72)	Yes
				LRTI: 0.79 (0.55‐1.11)	Length of hospital stay: **1.22 (1.03‐1.97)**	
Chaves, 2013 9	Hosp	<18	‐	‐	ICU admission or death: **2.19 (1.11‐4.33)**	Yes
		≤18	‐	‐	ICU admission or death: **2.21 (1.66‐2.94)**	Yes
Dangi, 2014 10	Non‐hosp	All ages	Any underlying condition: nr & ns	‐		Unclear
Cohen, 2014 30	Hosp	All ages	Any underlying condition: nr & ns	‐	ICU admission: nr & ns	Yes
					Mechanical ventilation: nr & ns	
					Oxygen supplementation: nr & ns	
					Length of hospital stay: nr & ns	
					Death: nr & ns	
Kusznierz, 2016 33	Hosp	All ages	‐	‐	ICU admission or death: **2.6 (1.0‐6.8)**	Yes
B Victoria vs B Yamagata						
Dangi, 2014 10	Non‐hosp	All ages	Any underlying condition: nr & ns	‐	‐	Unclear
Sočan, 2014 31	Non‐hosp	All ages	Chronic cardiovascular diseases: 0.8 (0.2‐2.7)	Pneumonia: 0.8 (0.2‐3.1)	‐	Yes
			Chronic respiratory diseases: 1.6 (0.1‐17.2)	Bronchitis: 1.2 (0.4‐3.4)		
			Diabetes mellitus: 0.7 (0.2‐2.7)			
			Chronic renal diseases: 1.3 (0.3‐6.0)			
			Obesity: 1.1 (0.2‐6.1)			

ns, not statistically significant; nr, not reported; Hosp, hospital‐based studies; Non‐hosp, non‐hospital‐based studies; ICU, intensive care unit; LRTI, lower respiratory tract infection; URTI, upper respiratory tract infection. Bold value indicates the significant findings (*p* < .05).

The majority of studies performed no adjustment for the patient's age (although some of them focused on specific age groups such as children,^7^, ^15^, ^16^ adults,^6^ or the elderly^17^) or other potential confounders. In these studies, proportions (for binary variables such as the presence/absence of signs and symptoms, underlying conditions, or complications) and mean/median values (for continuous variables such as the length of hospital stay) were reported and frequently compared using appropriate statistical tests. When no test was performed by the authors, we applied a large‐sample test to compare proportions, provided that the group‐specific sample size and proportions were reported by the study authors. The results of these studies were summarized in Tables S1 and S2 (for signs and symptoms) and Table S3 (for complications, outcomes, and underlying conditions).

## 3 RESULTS

**Figure 1:**
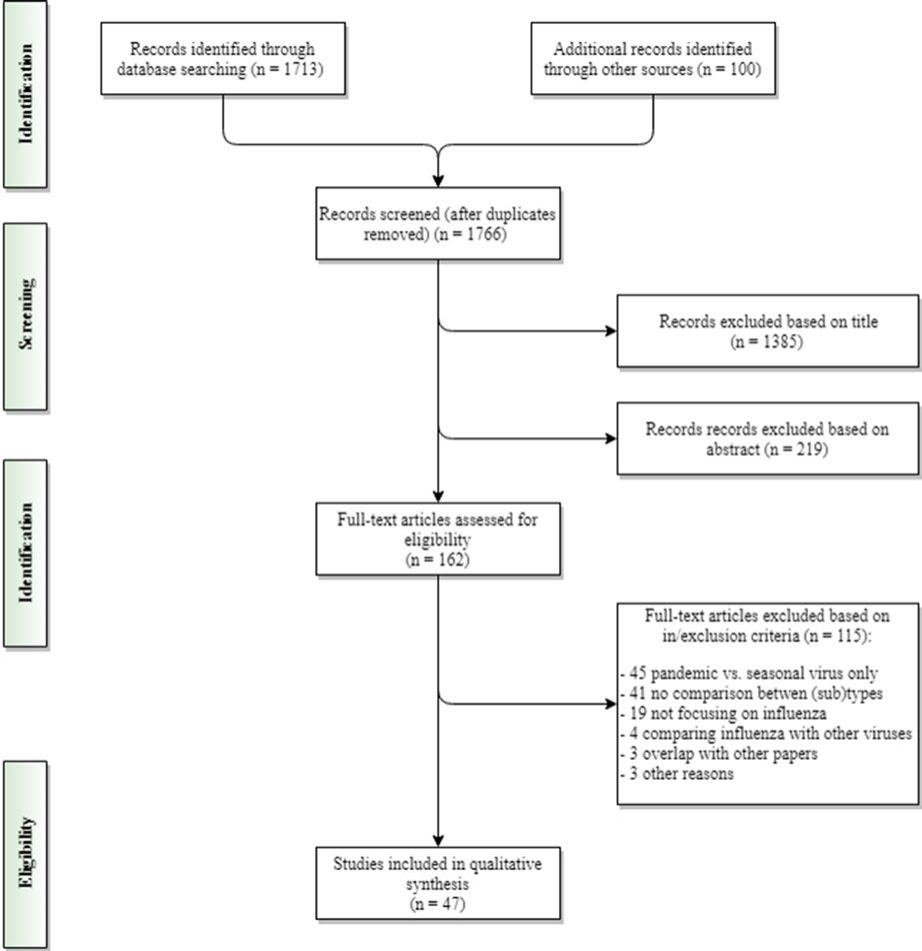
Flow diagram of the literature search

The literature search resulted in a total of 1766 titles as shown in the PRISMA flowchart (Figure 1), of which 1385 and 219 were excluded based on their title or abstract, respectively. The remaining 162 papers were obtained in full copy and assessed for eligibility. A total of 115 papers were excluded at this stage: The main reasons for exclusion were the fact that only a comparison of pandemic vs. unspecified non‐pandemic influenza virus was performed (n = 45), or there was no comparison between influenza virus (sub)types (n = 41). The literature review was therefore based on 47 independent papers (Figure 1).

An overview of the studies (Table 1, 9, 15, 17, 18, 19, 20, 21, 22, 23, 24, 25, 26, 27, 28, 29, 30, 31, 32, 33, 34, 35, 36 and Table 2, 6, 7, 8, 10, 11, 12, 16, 31, 37, 38, 39, 40, 41, 42, 43, 44, 45, 46, 47, 48, 49, 50, 51, 52, 53, 54) showed a lot of diversity in the populations that were investigated: Studies are presented in terms of hospitalized patients (n = 22) or cover mainly outpatient settings (n = 19), such as primary care (n = 6) or community‐based (n = 4) settings. Most studies were carried out in Asia (n = 17), Europe (n = 15), or North America (n = 8). The majority of studies were carried out from 2000 onwards (n = 41), and the number of subjects varied widely, from less than 100 to over 14 000. Patients of all ages were included in most studies (n = 22); 15 studies were limited to children and 10 studies to adults or elderly patients only. The main inclusion criteria for the patients were respiratory infections and symptoms (n = 15) or influenza‐like illness (n = 11). The influenza viruses that were most frequently compared were A(H1N1)p versus A(H3N2) (n = 23), influenza A versus B (n = 18), and A(H1N1) versus A(H3N2) (n = 12). The proportion of influenza patients that had received the vaccine was reported in 24 studies, but RR estimates were adjusted for vaccination status in only four papers.^8^, ^35^, ^51^, ^53^ Vaccinated patients were excluded from the analyses in four studies, and no or insufficient information on patients’ vaccination status was available in 19 studies. The use of antivirals by influenza patients was reported in 23 studies: Of these, only three^9^, ^27^, ^33^ provided RR estimates for antiviral use. In two studies,^6^, ^11^ influenza patients who received antiviral treatment were excluded from the analyses, while one study^51^ only included patients that received antiviral treatment. Finally, there was no or insufficient information on antiviral use in 21 studies.

The assessment of the quality of included studies is provided in the Data S1. Limitations common to most of the included studies were the following: lack of a sample size justification (or a precise calculation of the statistical power), poor clarity about how the outcome in the study was defined and assessed, and lack of adjustment for potential confounding (see below). Also, the participation rate and proportion of patients lost to follow‐up were not reported in many studies.

Only six papers reported odds ratios or risk ratios for differences in the frequency of symptoms and signs (Table 3, 10, 30, 31, 40, 41, 43). Overall hardly any significant differences were found between the different influenza viruses and when a significant result was found, no second study was found to support this finding. For the risk of fever, there were contradictory outcomes for A(H1N1)p vs. B. A similar overall finding was found for complications, and underlying conditions (Table 4, 8, 9, 10, 12, 19, 30, 31, 33, 40, 41, 44), with the only significant differences reported in three studies for A(H1N1)p versus A(H3N2), with different ICU admission rates or case‐fatality ratios (patients with A(H1N1)p were admitted more often to the ICU and died more often).

The assessment of the unadjusted differences in the frequency of symptoms and signs (Table S1, 15, 21, 22, 23, 24, 25, 26, 27, 28, 30, 31, 36 and Table S2, 6, 7, 8, 10, 11, 12, 16, 31, 37, 38, 39, 40, 41, 44, 46, 47, 48, 49, 50, 51, 53, 54) also showed few differences between the influenza viruses. Compared to influenza B, there was some evidence that patients with influenza A (not further specified) less often presented with myalgia (four studies—all focusing on children—of fifteen) were less often sent to the hospital for medical advice and/or further investigation (two studies of fourteen) and more often presented with cough (two studies of nine). With the exception of the finding for myalgia, there were no further age‐specific differences in the frequency of symptoms and signs between influenza viruses.

Concerning the frequency of complications and underlying conditions (Table S3, 9, 15, 17, 18, 19, 20, 21, 22, 23, 24, 25, 26, 27, 28, 29, 30, 31, 32, 33, 34, 35, 36), we also found very few significant differences between the influenza viruses in the unadjusted virus comparisons. There was some evidence that A(H1N1)p may result in more complications compared to other influenza virus (sub)types: People infected with A(H1N1)p more often had pneumonia and were more frequently admitted in the ICU compared to influenza B, and more frequently had upper respiratory tract infections, pneumonia, and ICU admissions compared to A(H3N2).

## 4 DISCUSSION

We aimed to assess the difference in clinical characteristics and illness severity for the different influenza virus types, subtypes, and lineages. Despite the common assumption that A(H3N2) infections result in more severe illness and that influenza B infections are milder, the current literature review did not reveal such differences. The association of a possible benign acute myositis with influenza B infection among children has been recognized^55^ and was confirmed in our review; except for this finding, the clinical differences between influenza viruses at disease onset were not large and frequently pointed in opposite directions for different studies. Likewise, the virus subtype did not seem to be a major determinant of severity, especially once the patient's age and pre‐existing health conditions were taken into account, with the possible exception for the A(H1N1)p virus subtype.

Knowing the virus type and subtype may help with the clinical management of a patient, and some researchers have stressed the importance of rapid testing tools to identify the type of virus,^40^ while, others have suggested that clinical relevance is low.^40^, ^43^ Our finding on A(H1N1)p suggests that the knowledge of the causal virus may be an important element for the clinician, as patients infected with this subtype deserve to be monitored more closely because of a higher risk of unfavorable outcomes. A recent paper (published after our literature search was closed) corroborated our findings by showing a higher ratios of death to hospitalization among the elderly (≥65 years) for A(H1N1)p compared to A(H3N2) and B influenza patients^56^; further well‐designed studies are needed to strengthen the evidence regarding this important point. The early identification of the causal virus type and subtype may also be useful when focusing on antiviral resistance^40^, ^43^ or bacterial co‐infections,^41^, ^44^ to promote a more prudent use of antiviral and antibacterial drugs. From a public health perspective, Yap and coll.^43^ have argued that it is important to know the clinical characteristics and severity of the different virus types and subtypes, because this information may help in the early detection of changes possibly indicating the emergence of a new (pandemic) virus strain. The early detection of new strains is important, as measures to prevent the spread of the new virus can be taken at an early stage. Information about the circulating viruses and their severity may also be important for communication purposes by public health authorities or to be better prepared for the impact of the seasonal epidemic (eg, in nursing homes and hospitals).

The studies included in our literature review showed a wide variety in design, populations, health seeking settings, and definitions, making it difficult to compare studies. Study populations could vary from all healthy persons to persons with certain conditions (eg, asthma) and from patients of any age to specific age groups (eg, children, adults). Settings varied from individuals seen by their practitioners, to patients visiting the emergency room of hospitals and clinics, and hospitalized patients. Even for similar health seeking settings, the characteristics of patients may vary because of differences in the healthcare system or patient pathway. For instance, in countries were general practitioners have a gate‐keeping function, a different selection of patients may go the hospital compared to countries where patients have direct access. Differences in health settings and healthcare access may also affect the delay of consultation and therefore further impact on the clinical presentation and severity of influenza patients. An additional source of diversity between studies may arise from different criteria being applied to select the patients that are swabbed. The definition of severity of illness also depends on the study population. For community‐based studies (and other studies not entirely based on hospitalized patients), the number of days of illness or the admission to the hospital was often chosen as an indicator of more severe illness. In contrast, the most common measures of severity in studies based on inpatients were the length of hospital stay, the frequency of admission to ICU, and in‐hospital death. This large diversity in populations, settings, and definitions may be a possible explanation of why significant results emerging from one study were very often not confirmed in subsequent studies.

A number of studies have found that influenza‐associated hospitalizations and deaths are highest in seasons dominated by A(H3N2),^3^, ^4^, ^5^ suggesting that the clinical presentation and severity of influenza may be worse for this subtype. However, we did not confirm this finding in our literature review. A number of factors may explain the higher burden linked with influenza A(H3N2) in these studies. A study carried out in England and Wales^57^ postulated that the influenza A(H3N2) virus, which emerged in 1968, has a “declining ability (…) to efficiently infect susceptible hosts” and was associated with very low ILI consultation rates after 2000 compared to earlier years. Our literature review mainly covered studies carried out after 2000, which was a period when influenza A(H3N2) may have been associated with lower relative severity compared to the other viruses. In addition, influenza virus types and subtypes tend to affect different age groups, with influenza A(H3N2) more frequently affecting the 65+ age group (Caini S, manuscript in preparation). The comparatively higher burden of disease associated with influenza A(H3N2) may be due to the greater susceptibility to this virus subtype of the elderly, as these represent the largest population at risk for severe and complicated influenza in industrialized countries.^58^

Our literature review has a number of limitations, which mostly originate from intrinsic limitations of the studies that were included. Most studies failed to control for potential confounding factors such as age, underlying condition(s), vaccine status, or antiviral treatment, as no multivariate analyses were performed. Frequently, this was not possible because of the relatively small numbers of influenza cases and some studies tried to overcome the lack of statistical power by combining all influenza A subtypes into one category. However, there was some evidence that the clinical outcomes of influenza illness could be worse for the A(H1N1)p strain; therefore, merging patients infected with different influenza A subtypes into one category may not be advisable. Likewise, combining data from the same setting over several consecutive seasons might be helpful to increase the number of study participants; however, the comparisons may be influenced in this case by the genetic drift of influenza viruses over time.^9^ Another limitation was that signs and symptoms may vary between mild and severe; therefore, their clinical presentation may not provide a precise measure of the severity of influenza (only a small number of studies made a distinction in the severity of signs and symptoms, for instance, by focusing on “high fever” instead of on fever in general). We did not focus on the age signature of the different influenza viruses in our review: However, some studies suggested that there is a difference between age groups affected by different influenza viruses,^8^, ^10^, ^30^, ^31^, ^33^, ^40^, ^41^, ^44^ and reviewing these data could provide additional knowledge. Another limitation of our review may lie in our search strategy. Studies were only searched in MEDLINE, and, although its coverage has been demonstrated to be generally high,^59^ some eligible papers were missed in the initial search. Concerning the search string, we used the Boolean operator OR several times to be as sensitive as possible in the earliest steps of the literature search; however, we were also forced to include “influenza” and “compare/d/s/comparison” in order to keep the number of screened entries to within reasonable limits, and some eligible papers may have also been missed because of this approach. The snowballing method revealed a significant number of additional papers and, while this increased the coverage of our search, we cannot rule out the possibility of having missed some studies.

In conclusion, we found very limited evidence that the different influenza virus types, subtypes, and lineages differ between one another in terms of clinical presentations, prevalence of underlying medical conditions, illness severity, or case‐fatality ratio. However, an important gap in knowledge still exists in this area, as drawing firm conclusions was made difficult by the low comparability and methodological limitations of many of the studies that were included. A minimum set of quality requirements for future studies on this topic should include a clear description of the study populations, settings, and in‐/exclusion criteria; a follow‐up of each patient during the entire illness course, that is, from onset until recovery or death (and including details of in‐hospital stay for patients that were hospitalized); and the use of multivariate regression techniques providing relative risk estimates adjusted by (at least) patient's age, underlying conditions, vaccine status, and antiviral treatment.

## CONFLICT OF INTEREST

Clotilde El Guerche‐Séblain is an employee of Sanofi Pasteur. Clotilde El Guerche‐Séblain is the scientific coordinator at Sanofi Pasteur of the research project, helped define the study objectives, and critically revised the manuscript. When reviewing the manuscript, the revisions did not concern the public health findings or conclusions. All the other authors declare they have no conflict of interest to disclose.

## AUTHORS’ CONTRIBUTIONS

JP, SC, and CEG‐S conceived the study. TW, JP, and SC extracted the data, performed the statistical analysis, and prepared the tables and figures. SC, JP, and MK wrote the first draft of the manuscript. All authors critically revised the manuscript and approved its final version.
