# Supplementary material for: Clinical characteristics and severity of influenza infections by virus type, subtype, and lineage: A systematic literature review
Source: Influenza Other Respir Viruses. 2018 Jul 20;12(6):780–92. doi: 10.1111/irv.12575 (PMC6185883; doi:10.1111/irv.12575)
Supplement: Supplementary file 2 [file IRV-12-780-s002.doc]

**Supplementary Table 1**. Frequency of selected signs and symptoms among patients infected with different virus types, subtypes and lineages, in hospital-based studies. Statistically significant differences are in bold.

| **First author, year** | **Age group or range** | **Fever ^(a)^** | **Headache** | **Myalgia** | **Malaise** | **Cough** | **Sore throat** | **Shortness of breath** |
| --- | --- | --- | --- | --- | --- | --- | --- | --- |
| **A vs. B** |  |  |  |  |  |  |  |  |
| Daley, 2000 [15] | children | 88% vs 82% | 1.6% vs 0% | – | – | 84% vs 78% | 1.6% vs 0% | – |
| Hu, 2004 [21] | children | 99% vs 97% | 10% vs 15% | – | – | 96% vs 91% | 8% vs 11% | – |
| Meury, 2004 [22] | children | **91% vs 86%** | – | – | – | 76% vs 57% | – | – |
| Mancinelli, 2016 [24] | children | **66% vs 44%** | – | – | – | – | – | – |
| Jennings, 2008 [26] | ≥18 | – | 56% vs 50% | **50% vs 100%** | – | 96% vs 83% | – | – |
| Loubet, 2016 [27] | ≥18 | 90% vs 88% | 26% vs 31% | 22% vs 24% | 28% vs 32% | 87% vs 85% | – | **72% vs 59%** |
| Seo, 2014 [28] | >19 | 100% vs 100% | – | – | – | 100% vs 100% | – | – |
| **A(H1N1)p vs. B** |  |  |  |  |  |  |  |  |
| Guan, 2015 [23] | children | 96% vs 90% | 8% vs 3% | 0 vs 9% | – | 100% vs 93% | – | – |
| Loubet, 2016 [27] | ≥18 | 92% vs 88% | 31% vs 31% | 27% vs 24% | 27% vs 32% | 88% vs 85% | – | **78% vs 59%** |
| **A(H3N2) vs. B** |  |  |  |  |  |  |  |  |
| Guan, 2015 [23] | children | 89% vs 90% | 2% vs 3% | **0% vs 8%** | – | 99% vs 93% | – | – |
| Loubet, 2016 [27] | ≥18 | 87% vs 88% | **19% vs 31%** | 19% vs 24% | – | 86% vs 85% | – | 67% vs 59% |
| Cohen, 2014 [30] | all ages | **50% vs 34%** | – | – | – | 97% vs 96% | – | – |
| **A(H1N1)p vs. A(H3N2)** |  |  |  |  |  |  |  |  |
| Guan, 2015 [23] | children | 96% vs 89% | 8% vs 2% | 0% vs 0% | – | 100% vs 99% | – | – |
| Yang, 2014 [25] | ≥14 | 98% vs 93% | 10% vs 17% | – | – | 97% vs 97% | – | 29% vs 27% |
| Loubet, 2016 [27] | ≥18 | 88% vs 86% | **31% vs 19%** | 27% vs 19% | 27% vs 29% | 88% vs 86% | – | **78% vs 67%** |
| **B Victoria vs. B Yamagata** |  |  |  |  |  |  |  |  |
| Tan, 2013 [36] | all ages | **81% vs 61%** | 71% vs 70% | 57% vs 61% | – | 29% vs 44% | 69% vs 79% | – |
| Sočan, 2014 [31] | all ages | 59% vs 41% | – | – | – | 58% vs 42% | – | 55% vs 45% |

^(a)^ Fever, high fever, feverishness
