# Supplementary material for: Clinical characteristics and severity of influenza infections by virus type, subtype, and lineage: A systematic literature review
Source: Influenza Other Respir Viruses. 2018 Jul 20;12(6):780–92. doi: 10.1111/irv.12575 (PMC6185883; doi:10.1111/irv.12575)
Supplement: Supplementary file 3 [file IRV-12-780-s003.doc]

**Supplementary Table 2**. Frequency of selected signs and symptoms, and proportion of hospitalization, among patients infected with different virus types, subtypes and lineages, in non-hospital-based studies. Statistically significant differences are in bold.

| **First author, year** | **Age group or range** | **Fever ^(a)^** | **Headache** | **Myalgia** | **Malaise** | **Cough** | **Sore throat** | **Shortness of breath** | **% sent to hospital** |
| --- | --- | --- | --- | --- | --- | --- | --- | --- | --- |
| **A vs. B** |  |  |  |  |  |  |  |  |  |
| Chi, 2008 [7] | children | 96% vs 100% | 12% vs 6% | 7% vs 9% | – | 91% vs 86% | – | – | – |
| Mosnier, 2015 [11] | 0-4 | 99% vs 99% | – | – | – | 78% vs 80% | – | – | – |
| Mosnier, 2015 [11] | 5-14 | 99% vs 99% | – | 57% vs 57% | – | **86% vs 82%** | – | – | – |
| Silvennoinen, 2009 [16] | ≤13 | ns & nr | ns & nr | ns & nr | – | ns & nr | ns & nr | – | – |
| Esposito, 2011b [37] | <14 | **60% vs 49%** | – | – | – | – | – | – | 0.8% vs 0.5% |
| Hong, 2015 [54] | <14 | **35% vs 44%** | 8% vs 7% | **1% vs 5%** | – | 80% vs 80% | 48% vs 50% | – | **5% vs 9%** |
| Shen, 2008 [38] | <16 | 98% vs 98% | 11% vs 15% | **11% vs 24%** | – | 93% 89% | 25% vs 35% | – | 29% vs 20% |
| Peltola, 2003 [39] | <17 | 94% vs 89% | – | **6% vs 15%** | – | 67% vs 60% | – | – | ns & nr |
| Hite, 2007 [40] | < 19 | 85% vs 91% | 15% vs 22% | **7% vs 18%** | – | 63% vs 77% | 13% vs 20% | 10% vs 16% | 33% vs 42% |
| Hong, 2015 [54] | ≥ 14 | 16% vs 20% | 35% vs 38% | 44% vs 37% | – | 88% vs 84% | 54% vs 57% | – | 18% vs 11% |
| Mosnier, 2015 [11] | 15-64 | 96% vs 97% | – | 89% vs 89% | – | 88% vs 90% | – | – | – |
| Mosnier, 2015 [11] | ≥65 | 96% vs 97% | – | 77% vs 85% | – | 89% vs 94% | – | – | – |
| Irving, 2012 [8] | all ages | ns & nr | ns & nr | ns & nr | – | **95% vs 79%** | **76% vs 66%** | – | ns & nr |
| Cohen, 2015 [53]  ^(b)^ | all ages | 99% vs 100% | 66% vs 65% | 60% vs 63% | – | 90% vs 92 | 56% vs 50% | – | 1% vs 1% |
|  |  | 87% vs 89% | 78% vs 74% | 78% vs 78% | – | 91% vs 95% | 45% vs 36% | – | **3% vs 13%** |
| **A(H1N1) vs. B** |  |  |  |  |  |  |  |  |  |
| Kaji, 2003 [6] | adults | 91% vs 86% | 49% vs 39% | **26% vs 52%** | 71% vs 73% | 19% vs 16% | **36% vs 14%** | – | – |
| Tang, 2010 [47] | all ages | 93% vs 83% | 21% vs 25% | 14% vs 33% | – | 79% v s 83% | 36% vs 25% | 7% vs 0% | – |
| Suess, 2012 [49] | all ages | 100% vs 100% | – | 100% vs 87% | – | 83% vs 90% | 67% vs 74% | – | – |
| **A(H1N1)p vs. B** |  |  |  |  |  |  |  |  |  |
| Gutiérrez-Pizarraya, 2012 [41] | >14 | **50% vs 28%** | – | – | – | – | – | – | 69% vs 58% |
| Mosnier, 2015 [11] | 0-4 | 99% vs 99% | – | – | – | 84% vs 78% | – | – | – |
| Mosnier, 2015 [11] | 5-14 | 99% vs 99% | – | 58% vs 57% | – | 90% vs 82% | – | – | – |
| Mosnier, 2015 [11] | 15-64 | 96% vs 97% | – | **87% vs 89%** | – | 93% vs 90% | – | – | – |
| Mosnier, 2015 [11] | ≥65 | 93% vs 97% | – | 85% vs 85% | – | 89% vs 94% | – | – | – |
| **A(H1N1)p vs. B Victoria** |  |  |  |  |  |  |  |  |  |
| Dangi, 2014 [10] | all ages | – | 49% vs 48% | **46% vs 28%** | – | 87% vs 90% | **57% vs 35%** | 11% vs 12% | – |
| **A(H1N1)p vs. B Yamagata** |  |  |  |  |  |  |  |  |  |
| Dangi, 2014 [10] | all ages | – | 49% vs 39% | 46% 41% | – | 87% vs 85% | 57% vs 48% | 11% vs 13% | – |
| **A(H3N2) vs. B** |  |  |  |  |  |  |  |  |  |
| Mosnier, 2015 [11] | 0-4 | 99% vs 99% | – | – | – | 80% vs 78% | – | – | – |
| Mosnier, 2015 [11] | 5-14 | 99% vs 99% | – | 57% vs 57% | – | 84% vs 82% | – | – | – |
| Mosnier, 2015 [11] | 15-64 | 97% vs 96% | – | 90% vs 89% | – | 87% vs 90% | – | – | – |
| Mosnier, 2015 [11] | ≥65 | 95% vs 97% | – | 75% vs 85% | – | 92% vs 94% | – | – | – |
| Wie, 2013 [44] | adults | – | **44% vs 55%** | 53% and 60% | **43% vs 55%** | 90% vs 90% | 57% vs 63% | – | **10% vs 25%** |
| Kaji, 2003 [6] | adults | **94% vs 86%** | 37% vs 39% | 50% vs 52% | 81% vs 73% | 18% vs 16% | **38% vs 14%** | – | – |
| Tang, 2010 [47] | all ages | 88% vs 83% | 9% vs 25% | 14% vs 33% | – | 81% vs 83% | 38% vs 25% | 7% vs 0% | – |
| Suess, 2012 [49] | all ages | 100% vs 100% | – | 88% vs 87% | – | 100% vs 90% | 88% vs 74% | – | – |
| Kawai, 2013 [51] | all ages | – | – | ns & nr | – | ns & nr | – | – | – |
| **A(H3N2) vs. B Victoria** |  |  |  |  |  |  |  |  |  |
| Dangi, 2014 [10] | all ages | – | 54% vs 47% | 35% vs 28% | – | **100% vs 90%** | 37% vs 32% | 21% vs 12% | – |
| **A(H3N2) vs. B Yamagata** |  |  |  |  |  |  |  |  |  |
| Dangi, 2014 [10] | all ages | – | **54% vs 39%** | 35% vs 41% | – | **100% vs 85%** | 37% vs 35% | 21% vs 13% | – |
| **A(H1N1) vs. A(H3N2)** |  |  |  |  |  |  |  |  |  |
| Esposito, 2011a [12] | <15 | **79% vs 91%** | – | – | – | – | – | – | **3.1% vs 16.3%** |
| Belongia, 2010 [46] | children and adolescents | 95% vs 96% | 72% vs 65% | 48% vs 50% | – | 97% vs 96% | 75% vs 80% | – | 3.7% vs 2.4% |
| Kaji, 2003 [6] | adults | 91% vs 94% | 49% vs 37% | **26% vs 50%** | 71% vs 81% | 19% vs 18% | 36% vs 38% | – | – |
| Belongia, 2010 [46] | adults | 87% vs 89% | 87% vs 83% | 80% vs 86% | – | 97% vs 99% | 81% vs 73% | – | 2.3% vs 4.2% |
| Lindblade, 2010 [48] | all ages | 100% vs 100% | 92% vs 88% | 81% vs 81% | – | 96% vs 86% | 92% vs 94% | 23% vs 25% | – |
| Tang, 2010 [47] | all ages | 93% vs 88% | 21% vs 9% | 14% vs 14% | – | 79% vs 81% | 36% vs 38% | 7% vs 7% | – |
| Suess, 2012 [49] | all ages | 100% vs 100% | – | 100% vs 88% | – | 83% vs 100% | 67% vs 88% | – | – |
| Yang, 2012 [50] | all ages | 21% va 22% | 64% vs 65% | 61% vs 56% | – | 50% vs 49% | 75% vs 81% | 3% vs 6% | – |
| **A(H1N1)p vs. A(H3N2)** |  |  |  |  |  |  |  |  |  |
| Dangi, 2014 [10] | all ages | – | 49% vs 54% | 46% vs 35% | – | **87% vs 100%** | **57% vs 37%** | 11% vs 21% | – |
| **B Victoria vs. B Yamagata** |  |  |  |  |  |  |  |  |  |
| Chi, 2008 [7] | children | 100% vs 100% | 2% vs 13% | 6% vs 13% | – | 89% vs 100% | – | – | – |
| Mosnier, 2015 [11] | 0-4 | 100% vs 100% | – | 29% vs 25% | – | 82% vs 80% | – | – | – |
| Mosnier, 2015 [11] | 5-14 | 99% vs 99% | – | 58% vs 53% | – | 85% vs 81% | – | – | – |
| Mosnier, 2015 [11] | 15-64 | 97% vs 96% | – | 88% vs 90% | – | 90% vs 89% | – | – | – |
| Mosnier, 2015 [11] | ≥65 | 100% vs 94% | – | 92% vs 83% | – | 92% vs 97% | – | – | – |
| Dangi, 2014 [10] | all ages | – | 47% vs 39% | 28% vs 41% | – | 90% vs 85% | 32% vs 35% | 12% vs 13% | – |
| Sočan, 2014 [31] | all ages | 60% vs 40% | 60% vs 40% | 56% vs 44% | – | 60% vs 40% | 58% vs 42% | – | – |

ns: not significant

nr: not reported

^(a)^ Fever, high fever, feverishness

^(b)^ Results were reported separately for France (upper row) and Turkey (lower row)
