# Supplementary material for: Clinical characteristics and severity of influenza infections by virus type, subtype, and lineage: A systematic literature review
Source: Influenza Other Respir Viruses. 2018 Jul 20;12(6):780–92. doi: 10.1111/irv.12575 (PMC6185883; doi:10.1111/irv.12575)
Supplement: Supplementary file 4 [file IRV-12-780-s004.doc]

**Supplementary Table 3**. Frequency of selected complications, length of hospitalization, ICU admission, case-fatality rate, and underlying conditions, among patients infected with different virus types, subtypes and lineages, in hospital-based studies. Statistically significant differences are in bold.

| **First author, year** | **Age group or range** | **Complications** | | | | **Hospitalization and length of stay** | **ICU admission** | **Case fatality rate** | **Underlying conditions** |
| --- | --- | --- | --- | --- | --- | --- | --- | --- | --- |
|  |  | **URTI** | **Bronchitis or bronchiolitis** | **Pneumonia** | **Other** |  |  |  |  |
| **A vs B** |  |  |  |  |  |  |  |  |  |
| Weigl, 2002 [18] | ≤16 | 51% vs 14% | 12% vs 29% | 25% vs 36% | 11% vs 14% (wheezing bronchitis) | (no test performed) | – | – | With a condition: **18% vs 43%** |
| Dawood, 2011 [19] | ≤17 | – | – | 39% vs 40% | Asthma exacerbation: **50% vs 29%** | – | 17% vs 15% | – | Any (besides asthma): **26% vs 42%** |
| Daley, 2000 [15] | children | 16% vs 19% | 22% vs 15% | 19% vs 19% | – | Mean no. days: 4.8 vs 4.0 (no test performed) | 8% vs 11% | – | Any underlying condition: **39% vs 70%** |
| Hu, 2004 [21] | children | 52% vs 64% | 8% vs 6% | **21% vs 7%** | Myositis: **6% vs 34%** | – | – | – | – |
| Meury, 2004 [22] | children | 57% vs 50% | 7% vs 21% | 21% vs 7% | Croup: 5% vs 7% | Mean no. days: 4 vs 3 (ns) | 5% vs 0% | 0% vs 0% | Any: 26% vs 29% |
| Mancinelli, 2016 [24] | children | – | 6% vs 8% | 11% vs 15% | – | Median no. days: 5 vs 3 (no test performed) | – | – | Underlying disease: 24% vs 14% |
| Jennings, 2008 [26] | ≥18 | – | – | – | – |  | 9% vs 0% | 9% vs 0% | Asthma: 13% vs 0% Heart failure: 13% vs 17% Cerebrovacular disease: 13% vs 0% COPD: 35% vs 17% Diabetes: 13% vs 17% Renal disease: 4% vs 17% |
| Loubet, 2016 [27] | ≥18 | – | – | 24% vs 30% | Acute heart failure: 13% vs 9% ARDS: 11% vs. 8% Respiratory failure: 22% vs. 17% | Median no. days: 6 vs 5 (no test performed) | 16% vs 11% | 3% vs 4% | Chronic respiratory disease: **44% vs 31% C**hronic heart disease: 41% vs 35% Chronic renal failure: 11% vs 13% Diabetes: 25% vs 23% |
| Seo, 2014 [28] | >19 | – | – | – | – | Mean no. days: 6.9 vs 7.5 (no test performed) | 13% vs 10% | 6% vs 7% | Asthma: 4% vs 3% Cerebrovascular disease: 7% vs 3% Chronic renal disease: 9% vs 7% COPD: 16% vs 10% Diabetes: 15% vs 16% Heart failure: 6% vs 13% Malignancy: 15% vs 16% |
| Rahamat-Langendoen, 2012 [29] | all ages | – | – | – | Any complication: 38% vs 46% | – | 26% vs 31% | – | Underlying illness: 87% vs 77% |
| Drinka, 1999 [17] | elderly | – | – | – | – | – | – | 5% vs 5% | – |
| **A(H1N1)p vs B** |  |  |  |  |  |  |  |  |  |
| Chaves, 2013 [9] | children | – | 11% vs 11% | **29% vs 21%** | ARDS: 3% vs 2% | – | **22% vs 15%** | 0% vs 0% | Underlying condition: 41 vs 45% |
| Guan, 2015 [23] | children | – | 23% vs 36% | 77% vs 64% | Any complication: 15% vs 22% | Mean no. days: 8.7 vs 9.1 (ns) | – | – | – |
| Chaves, 2013 [9] | ≥18 | – | 0% vs 0% | **39% vs 32%** | ARDS: **6% vs <1%** | – | **25% vs 16%** | 4% vs 4% | Any underlying condition: 88% vs 87% |
| Loubet, 2016 [27] | ≥18 | – | – | 23% vs 30% | ARDS: 15% vs 8% Acute heart failure: 13% vs 9% Respiratory failure: 21% vs 17% | – | 18% vs 11% | 2% vs 4% | Chronic heart disease: 41% vs 35% Chronic renal failure: 8% vs 13% Chronic respiratory disease: 34% vs 31% Diabetes: 18% vs 23% |
| **A(H1N1)p vs B Yamagata** |  |  |  |  |  |  |  |  |  |
| Puig-Barberà, 2016a [34] | all ages | 12% vs 8% | – | **26% vs 13%** | COPD exacerbation: **9% vs 3%** | – | **0% vs 4%** | 2% vs 0% | Cardiovascular disease: **23% vs 12%** COPD: **22% vs 12%** Diabetes: **19% vs 5%** Asthma: 9% vs 9% Chronic renal disease: 8% vs 4% Malignancy: 4% vs 0% |
| Puig-Barberà, 2016b [35] | all ages | **23% vs 14%** | 23% vs 14% | 13% vs 12% | 16% vs 17% | – | – | – | Cardiovascular disease: 16% vs 16% COPD: 6% vs 9% Diabetes: 7% vs 5% Asthma: 4% vs 4% Chronic renal disease: 2% vs 4% Malignancy: 3% vs 3% |
| **A(H3N2) vs B** |  |  |  |  |  |  |  |  |  |
| Guan, 2015 [23] | children | – | 29% vs 36% | 71% vs 64% | Any complication: 26% vs 22% | Mean no. days: 10.0 vs 9.1 (ns) | – | – | – |
| Chaves, 2013 [9] | children | – | 10% vs 11% | 16% vs 21% | ARDS: 1% vs 2% | – | 1% vs 2% | 0% vs 0% | Underlying condition: 42 vs 45% |
|  | ≥18 | – | 0% vs 0% | 33% vs 32% | ARDS: 2% vs 2% | – | 14% vs 16% | 4% vs 4% | Any underlying condition: 87% vs 87% |
| Loubet, 2016 [27] | ≥18 | – | – | 23% vs 30% | Acute heart failure: 12% vs 9% ARDS: 8% vs 8% Respiratory failure: 21% vs. 17% | – | 14% vs 11% | 4% vs 4% | Chronic respiratory disease: **45% vs 31%** Chronic heart disease: **47% vs 35%** Chronic renal failure, diabetes: 14% vs 13% |
| Cohen, 2014 [30] | all ages | – | – | – | – | % staying ≥2 days: **71% vs 79%** | 1% vs 0% | 3% vs 4% | Any underlying medical condition: 7% vs 9% |
| Ishiguro, 2016 [32] | all ages | – | – | – | Respiratory failure: 59% vs 48% | – | – | 3% vs 7% | Pulmonary disease: 62% vs 50% Any ystemic disease: 44% vs 41% |
| **A(H3N2) vs B Yamagata** |  |  |  |  |  |  |  |  |  |
| Puig-Barberà, 2016a [34] | all ages | 4% vs 8% | – | 14% vs 13% | COPD exacerbation: **1% vs 3%** | – | **3% vs 0%** | 1% vs 0% | Cardiovascular disease: 18% vs 12% COPD: 9% vs 12% Diabetes: 6% vs 5% Asthma: 7% vs 9% Chronic renal disease: 5% vs 4% Malignancy: 3% vs 0% |
| Puig-Barberà, 2016b [35] | all ages | **10% vs 14%** | **7% vs 12%** | 17% vs 17% | COPD exacerbation: **5% vs 2%** | – | – | – | Cardiovascular disease: **26% vs 16%** COPD: **18% vs 9%** Diabetes: **15% vs 5%** Asthma: 5% vs 4% Chronic renal disease: 9% vs 4% Malignancy: 3% vs 3% |
| **A(H1N1) vs A(H3N2)** |  |  |  |  |  |  |  |  |  |
| Chiu, 2011 [20] | <18 | 51% vs 53% | – | 10% vs 11% | – | Median no. days: 3 vs 2 (no test performed) | – | – | % with ≥1 underlying conditions: 23% vs 23% |
| **A(H1N1)p vs A(H3N2)** |  |  |  |  |  |  |  |  |  |
| Chaves, 2013 [9] | children | – | 11% vs 10% | **29% vs 16%** | ARDS: 3% vs 1% | – | **22% vs 13%** | 0% vs 0% | Any underlying condition: 40% vs 39% |
| Guan, 2015 [23] | children | – | 23% vs 29% | 77% vs 71% | Any complications: 15% vs 26% | Mean no. days: 8.7 vs 10.0 (ns) | – | – | – |
| Yang, 2014 [25] | ≥14 | – | – | – | – | 81% vs 57% | 2% vs 0% | 2% vs 0% | Any underlying condition: **33% vs 10%** |
| Chaves, 2013 [9] | ≥18 | – | 0% vs 0% | **39% vs 33%** | **6% vs 2%** | – | **25% vs 13%** | 4% vs 4% | Any underlying condition: 88% vs 87% |
| Kusznierz, 2016 [33] | all ages | – | – | 59% vs 52% | Any complication: **56% vs 31%** | Median no. days: 5 vs 7 (no test performed) | **65% vs 35%** | 22% vs 15% | Any underlying condition: 76% vs 79% |
| Loubet, 2016 [27] | ≥18 | – | – | 23% vs 23% | ARDS: **15% vs 8%** | – | 18% vs 14% | 4% vs 2% | Chronic renal failure: **8% vs 14%** Chronic respiratory disease: **34% vs 45%** Diabetes: **18% vs 30%** Chronic heart disease: 41% vs 47% |
| Puig-Barberà, 2016a [34] | all ages | **12% vs 4%** | – | **26% vs 14%** | COPD exacerbation: **9% vs 1%** | – | 4% vs 0% | 2% vs 1% | Cardiovascular disease: 23% vs 18% COPD: **22% vs 9%** Diabetes: **19% vs 6%** Asthma: 9% vs 7% Chronic renal disease: 8% vs 5% Malignancy: 4% vs 3% |
| Puig-Barberà, 2016b [35] | all ages | **23% vs 10%** | **13% vs 7%** | 16% vs 17% | COPD exacerbation: **3% vs 5%** | – | – | – | Cardiovascular disease: **16% vs 26%** COPD: **6% vs 18%** Diabetes: **7% vs 15%** Asthma: 4% vs 5% Chronic renal disease: **2% vs 9%** Malignancy: 3% vs 3% |
| **B Victoria vs B Yamagata** |  |  |  |  |  |  |  |  |  |
| Tan, 2013 [36] | all ages | 81% vs 76% | – | – | – | – | – | – | – |

ns: not significant

ARDS: acute respiratory distress syndrome

COPD: chronic obstructive pulmonary disease
